# Performance analysis of different surface reconstruction algorithms for 3D reconstruction of outdoor objects from their digital images

**DOI:** 10.1186/s40064-016-2425-9

**Published:** 2016-06-30

**Authors:** Abhik Maiti, Debashish Chakravarty

**Affiliations:** Department of Mining Engineering, Indian Institute of Technology, Midnapur West, Kharagpur, West Bengal 721302 India

**Keywords:** 3D reconstruction, Poisson surface reconstruction, Ball-pivoting algorithm, Photogrammetry, Point cloud

## Abstract

3D reconstruction of geo-objects from their digital images is a time-efficient and convenient way of studying the structural features of the object being modelled. This paper presents a 3D reconstruction methodology which can be used to generate photo-realistic 3D watertight surface of different irregular shaped objects, from digital image sequences of the objects. The 3D reconstruction approach described here is robust, simplistic and can be readily used in reconstructing watertight 3D surface of any object from its digital image sequence. Here, digital images of different objects are used to build sparse, followed by dense 3D point clouds of the objects. These image-obtained point clouds are then used for generation of photo-realistic 3D surfaces, using different surface reconstruction algorithms such as Poisson reconstruction and Ball-pivoting algorithm. Different control parameters of these algorithms are identified, which affect the quality and computation time of the reconstructed 3D surface. The effects of these control parameters in generation of 3D surface from point clouds of different density are studied. It is shown that the reconstructed surface quality of Poisson reconstruction depends on *Samples per node* (SN) significantly, greater SN values resulting in better quality surfaces. Also, the quality of the 3D surface generated using Ball-Pivoting algorithm is found to be highly depend upon *Clustering radius* and *Angle threshold* values. The results obtained from this study give the readers of the article a valuable insight into the effects of different control parameters on determining the reconstructed surface quality.

**Electronic supplementary material:**

The online version of this article (doi:10.1186/s40064-016-2425-9) contains supplementary material, which is available to authorized users.

## Background

Three-dimensional modeling of geo-objects (like rock mass, underground tunnel, etc.) plays an important role in various fields of engineering, like rock mechanics, geology, civil and mining. Some of the existing methods for 3D modeling of geo-objects include models generated from laser scanner, CAD based 3D modeling of underground tunnels and traditional surveying. However, CAD-based modelling is a completely manual process. Modelling of a large-scale environment using CAD-based modelling softwares is extremely difficult, mainly due to the irregular geometries of the objects present in the scene being modelled.

Laser-scanner based 3D reconstruction of rock-face is fast and accurate; it also gives photo-realistic textured 3D models of a scene or object. However, as laser scanners are very expensive, they are not widely used to study the geo-mechanical characteristics of rocks, underground tunnels, etc. These problems in the existing 3D modelling techniques have inspired researchers in using photogrammetry to create photo-realistic 3D objects from digital images of different scenes. 3D reconstruction using digital images can be used for mapping and photo-realistic modelling of outdoor geo-objects like rock mass. As digital cameras and computers are pretty common in households and industries these days, the cost of image-based reconstruction process is negligible.

In this paper, a 3D surface reconstruction framework is discussed using which photorealistic 3D models of irregular shaped rock mass and other geo-objects can be obtained, using digital images of the objects. Using correspondence matching across the digital image sequences, a dense 3D point cloud of the geo-object is obtained. Then, surface reconstruction algorithms are run on the point cloud to generate a watertight surface over those points. The Poisson Surface Reconstruction and Ball-Pivoting algorithms for surface reconstruction are used in several dense point cloud of geo-objects (obtained from digital image sequence), changing the parameters affecting the reconstruction process. The effects of these control parameters on the quality of the generated surface are studied. The variation of computation time with respect to the control parameters is also studied.

## Related work

3D surface reconstruction from a digital image sequence of a scene or object is a challenging and important task in computer vision. Several surface reconstruction algorithms have been used by different authors over the past decade, in order to get a photo-realistic and accurate surface reconstruction from image sequences of different objects.

Boissonnat (1984) proposed an algorithm based on tangent plane estimation. In this algorithm, for a given point cloud, the neighborhood of each point is selected. The selected points are then projected on a tangent plane. Tangent planes are computed for each point in the point cloud using Delaunay triangulation. From these tangent planes, the approximate surface surrounding the point cloud can be found out. However, this method is computationally exhaustive, as the tangent plane needs to be calculated at each point in the cloud. Also, this algorithm is highly susceptible to noise.

The approach proposed by Hoppe et al. (1992) works on principle of tangent plane estimation and tracking of contours, generated from point clouds. Here, principle component analysis (PCA) is used for estimation of tangent planes. The direction vectors of the normals of the tangent planes are found out by estimating the eigenvectors generated from PCA. Then, the marching cubes algorithm is used to extract surface from 3D points. However, this algorithm fails where the point clouds are of low density. This is because tangent planes can’t be accurately estimated for sparse point cloud.

Delaunay triangulation/Voronoi diagram based reconstruction is another popular reconstruction approach, used by many researchers in this field. Voronoi-based surface reconstruction algorithm was first proposed by Amenta et al. (1998). The Crust algorithm, as described by Amenta et al. (2000), computes the Voronoi diagram of the points in the point cloud. Then, Delaunay Triangulation is computed using the Voronoi diagrams over the point cloud. A 3D crust is reconstructed by obtaining the all triangles connecting three points from the Delaunay triangulation. The time complexity of this algorithm is O (n log n).

The Tight Cocone Algorithm proposed by Dey and Giesen (2002) uses Cocone triangles (Amenta et al. 2000) of the point cloud. After computation of Cocone triangles, tetrahedral triangles are marked as ‘in’ or ‘out’. The reconstructed surface is generated by joining the triangles marked as ‘in’. This algorithm has a computational complexity of O (n^2^). Also, this algorithm is not robust enough to handle noisy and low density point cloud.

The main idea behind the Power Crust algorithm (Amenta et al. 2001) is an approximation of the reconstructed surface, taking the union of median balls. The surface is represented as a polygon, obtained from the polar balls using the Voronoi diagram. This algorithm is robust as it is expressed in terms of boundary cells of a solid object. No hole-filling mechanism is required in this surface reconstruction algorithm, as it captures the actual geometry of the surface. However, noisy data cause scattering of points away from the surface.

The Ball-Pivoting algorithm (BPA) (Bernardini et al. 1999) is used for surface reconstruction from a dense point cloud. A seed triangle is selected as the starting point of the mess generation. A ball of predefined radius is then pivoted along an edge of the triangle and rolled through the point cloud until it touches another point in the point cloud. The newly found point is joined with the pivoting edges to form another triangle. As the ball rolls through the entire point cloud, a 3D mesh is formed, joining the triangles that are created by the rolling ball. This 3D mesh is the output of the BPA algorithm. As the ball is pivoted until all the points in the point cloud is scanned, this process is data driven and sensitive to noise.

The ball-pivoting takes a lot of time to complete and is memory intensive. So, parallel processing of this algorithm was implemented for this algorithm in by Digne (2014) in his paper. For point cloud of varying density, BPA was done with multiple ball radii. A hole-filling algorithm was implemented for portions of point cloud having low-density. Also, adaptive ball radius was used for reconstructing point clouds of non-uniform density. Ball radius was taken as a function of sampling density of the point cloud.

Poisson surface reconstruction (Kazhdan et al. 2006) forms a Poisson equation for solving the best-fit surface of a dense point cloud. In this approach, point clouds with oriented normal are required as input. An indicator function is defined whose value is one inside and zero outside the reconstructed. The gradient of the indicator function is then equated to a vector field, built from the point cloud normal vectors. Then, Poisson equation is formed and is solved to get the indicator function. The watertight surface is reconstructed using the marching cubes algorithm and stored in an octree.

In original Poisson reconstruction approach (Kazhdan et al. 2006), all points in a point cloud were used to extract the reconstructed surface. Kazhdan and Hoppe (2013) extended the Poisson surface reconstruction technique, incorporating sample weight values assigned for interpolation of missing points. Li et al. (2010) proposed an improvement to the Poisson reconstruction algorithm. The marching cube (MC) algorithm, used for surface extraction from indicator function, was improved to use interpolation and fast searching between points in the point cloud. This modification made the surface reconstruction computationally efficient. Also, the reconstructed surface came out to be more detailed.

## Methods

**Fig. 1:**
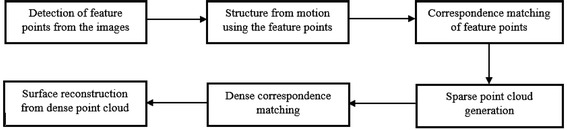
Flow diagram of the surface reconstruction process

**Fig. 2:**
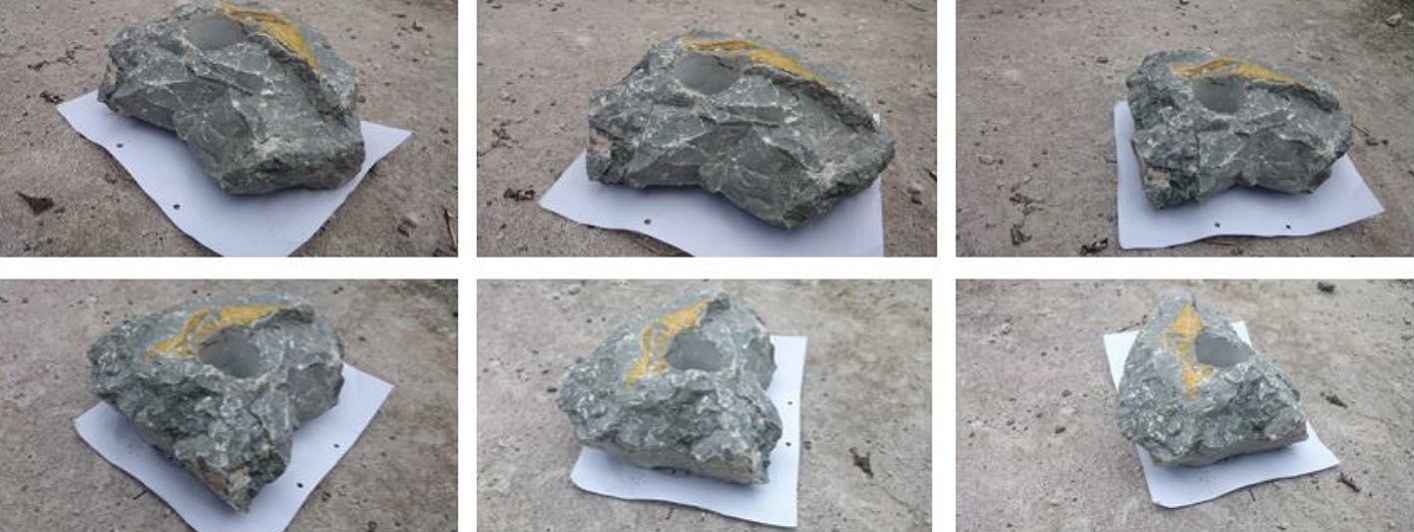
Image sequence of a rock-mass

**Fig. 3:**
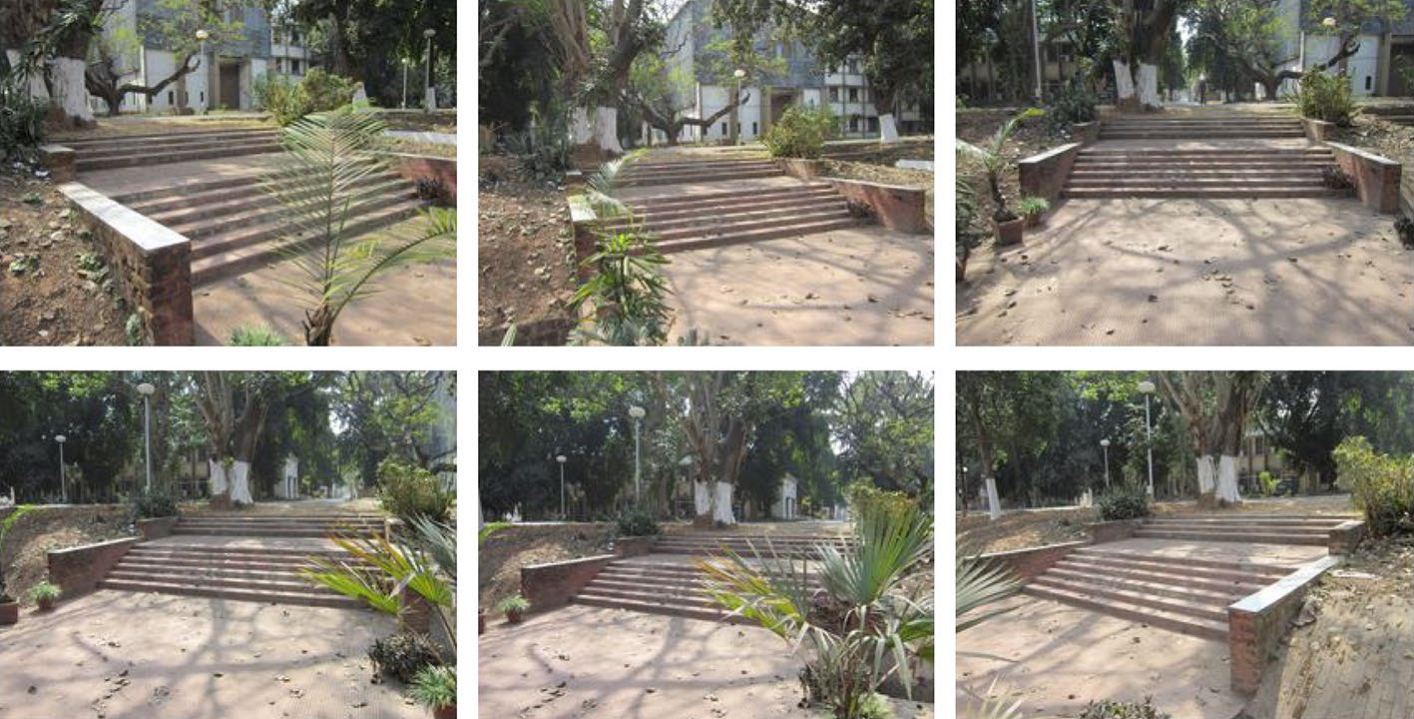
Image sequence of a stair case

The methodology applied for 3D surface reconstruction of geo-objects from a digital image sequence is pictorially described in Fig. 1. The main input of the surface reconstruction process is a digital image sequence of the scene or object being reconstructed. Figures 2 and 3 shows some images of the two image sequences, which were used as inputs in this paper. The basic concepts of surface reconstruction from uncalibrated image sequence can be found in (Koch et al. 2000).

### Detection of feature points

The first step in 3D reconstruction of an object from its digital images is correspondence matching. Corresponding points refer to those points across the images, which are the projection of a same 3D point of the object being imaged. For correspondence matching, a set of distinctive feature-points are detected in each image. These feature points are searched for correspondence across the rest of the images. All the feature points are then matched across the images, for the correspondence matching to be complete.

Detecting the feature points in an image reduces the number of points to be matched for correspondence. Exhaustive matching of all the pixels of an image with the pixels of another image is computationally expensive. Also, the robust feature points are readily distinguishable and invariant to image transformations. Thus, the computation time of correspondence matching is greatly reduced by detecting feature points in the images. SIFT (Scale-Invariant Feature Transform) algorithm is used for detection of feature-points in an image.

### Structure from motion

Structure from motion (Sfm) is an iterative process of calculating the 3D positions of the corresponding matched points along with the camera parameters, from a set of matched points in an image sequence. This process is successfully implemented in sparse point cloud building from large image sequences, as has been described in (Agarwal et al. 2009).

#### Correspondence matching of feature points

The first task in structure-from-motion process is finding out the correspondence matches for the SIFT detected feature points, across the images. Here, approximate nearest neighbor search is used for finding out the matched points as algorithms like normalized cross correlation (NCC) and brute force method are computationally expensive, hence infeasible. Approximate nearest neighbor (ANN) search uses the three dimensional k-d tree data structure for implementation of correspondence matching. The following steps are followed in feature matching between images:

- An image pair, having some overlapping portion of the scene/object between them, is selected from the image sequence.
- All the feature points from one image are inserted into the leaves of the k-d tree. The feature points belonging to the other image of the pair are used as queries to the first image. The complexity of building the k-d tree in O (n.log n).
- Then, the k-d tree is used for efficient search of K-nearest neighbor of a feature point X. The threshold distance R is selected according to the image resolution. The time complexity of this search is ~O (log n).
- This search across the k-d tree leaves result in correspondence matching of the feature points in the image pair.
- The matched points are then checked for accuracy using RANSAC based estimation of the fundamental matrix. Those matched points, which are not satisfying the fundamental matrix equation, are rejected as mismatches.

#### Sparse point cloud building using bundle adjustment

After finding out the matched points across the image sequence, all sets of matched point sets are triangulated to retrieve the 3D location of the points in the object. This triangulation process builds a three-dimensional point cloud in space, each point belonging to some portion of the object/scene being reconstructed. This triangulation process is done using Bundle Adjustment (BA) method.

**Fig. 4:**
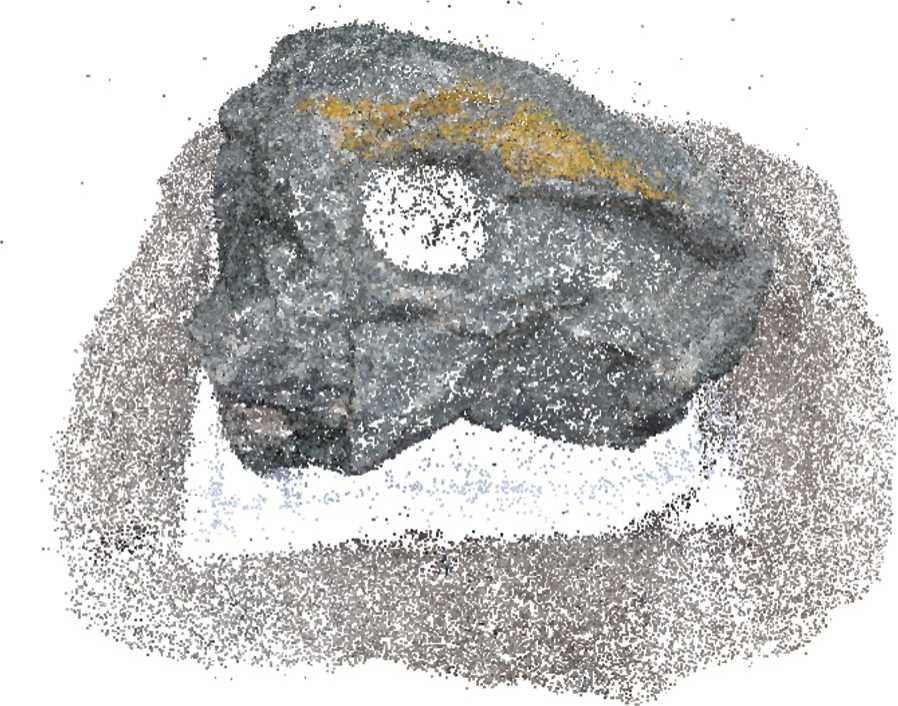
Sparse point cloud of a rock mass

**Fig. 5:**
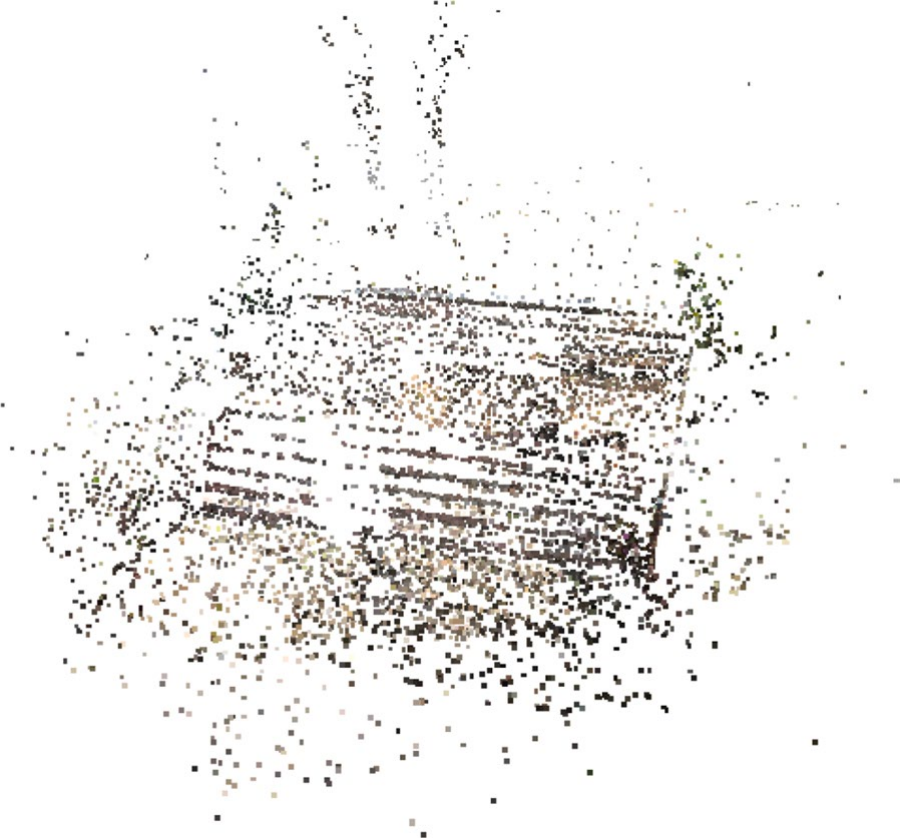
Sparse point cloud of a stair case

Bundle adjustment is a non-linear least-square optimization technique. A number of authors have used this optimization technique, including Lhuillier and Quan (2005), Agarwal et al. (2009). It is used to add and refine triangulated 3D points in an iterative approach by estimation of camera poses and relative motion between the image frames. BA uses a cost function to minimize the reprojection error between the 3D point triangulated by structure-from-motion and the observed location of the 3D point. After a 3D point is triangulated from a corresponding point pair, several more images are added to the system having the same sets of corresponding points. All the matched points are iteratively added to the triangulation. The error being propagated by triangulation is minimized as more and more matched points are used to triangulate a 3D point. Sparse point cloud generated from structure-from-motion process is shown in Figs. 4 and 5.

### Dense correspondence matching

Sparse point cloud generated using sfm only have 3D coordinates of SIFT identified feature points in it. The resulting point cloud is of low density and is inappropriate for reconstruction, as it does not contain finer details of the object being reconstructed. Hence, dense correspondence matching is needed to obtain a dense point cloud. While interpolating the sparse point cloud to build the dense point cloud can be done, several finer details in the object are missed out during the process, and object geometry cannot be retained exactly by interpolation methods. In dense correspondence matching, all the points in the image are searched for corresponding points across the image sequence. The dense correspondence matching process, as described in (Alcantarilla et al. 2013), gives a dense 3D point cloud from a large image sequences, accurately describing the geometry of the scene or object being reconstructed.

As there are several million pixels in a standard digital camera image, exhaustive search for all the pixels in the images are computationally exhaustive and thus infeasible. The following steps are undergone during a dense stereo matching:

- A few image frames with minimal camera motion is selected from the image sequence. By doing this, image redundancy is addressed as image frames having large camera motion between them have less amount of overlapping object regions. So, adding two frames with large camera motion does not give many corresponding points.
- Dense stereo matching is run in these camera frames to obtain a dense 3D point cloud of the overlapping object region. Real-time plane sweeping algorithm is used in each of the image frames during stereo matching. The basic idea behind this process is epipolar geometry. Two corresponding points in a stereo setup follow an epipolar constraint, because of which, the corresponding point in an image frame will lie along the epipolar line. This constraint reduces the correspondence point search area from 2-D plane across the entire image, to a one dimensional epipolar line existing in the stereo image pair counterpart.
- Real-time plane-sweeping method is implemented by sweeping a plane through 3D space, across the image frames following the camera positions. Light rays from all the pixels of the images are projected into the respective imaging planes.
- These rays, when back projected towards the object/scene being reconstructed, intersect each other at 3D points. These points come from all the points across the images. So, the intersection of these rays results in a dense collection of points in 3D space, which represent the object/scene being imaged. The obtained point cloud preserves the object geometry and form a dense 3D point cloud.

**Fig. 6:**
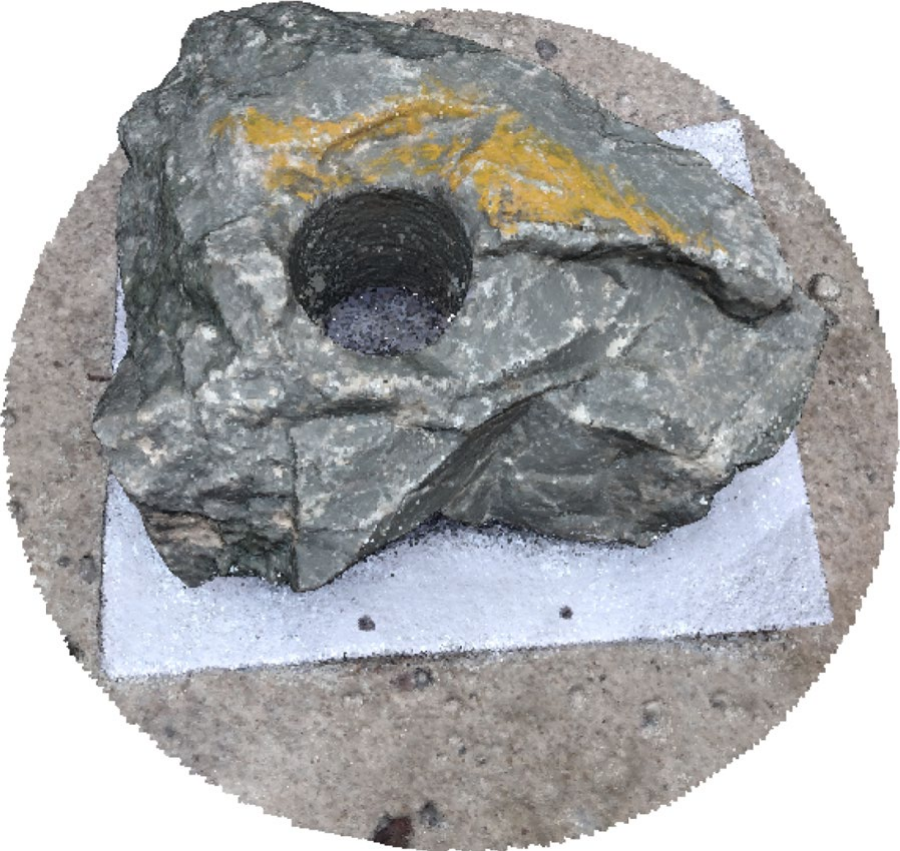
Dense point cloud of the rock mass

**Fig. 7:**
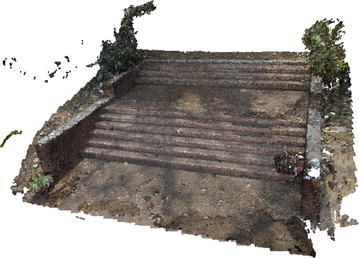
Dense point cloud of the stair case

Figures 6 and 7 show dense point cloud obtained through the above process, from sparse point clouds in Figs. 4 and 5. The point cloud stores the 3D (X, Y, Z) coordinates and preserve RGB color information. Additionally it has normal vectors (n_x_, n_y_, n_z_) at each of the 3D points.

### Generation of 3D watertight surface from dense 3D point cloud

After obtaining the 3D dense point cloud, 3D watertight surface needs to be constructed, over the point cloud. For construction of surface over a 3D dense point cloud, the point cloud needs to have 3D coordinates (X, Y, Z) and surface normals (n_x_, n_y_, n_z_) at each point. The surface normals for each point in a point cloud may be calculated from its (X, Y, Z) coordinates using principal component analysis (PCA). PCA is used to find out the eigenvector of a point in the point cloud over its local neighborhood. Additionally, the dense point cloud generated from image sequence has color information (RGB) attached to each of the points. A number of existing surface reconstruction algorithms have been tested over the point cloud obtained from digital images. The performance analysis of these reconstructions algorithms are done w.r.t time, resource usage, quality of reconstruction, etc. Brief overviews of these algorithms are given in the following section.

#### Poisson surface reconstruction

**Fig. 8:**
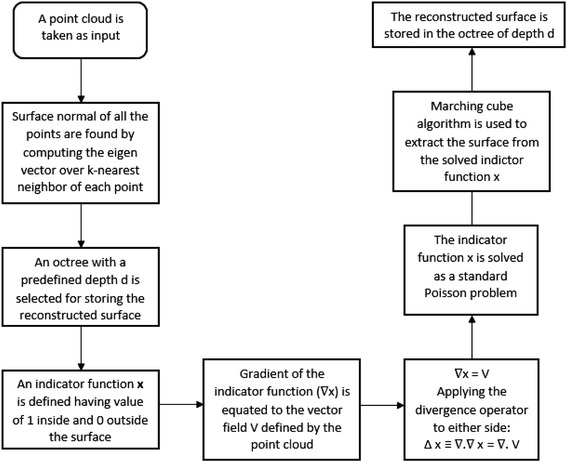
Flowchart of Poisson surface reconstruction

Poisson surface reconstruction aims at creating a 3D mesh from a dense point cloud, by minimizing the difference between the surface normal directions of the reconstructed surface and the 3D points in the point cloud. The algorithm is described in details in (Kazhdan et al. 2006). A flowchart of the Poisson reconstruction algorithm is given in Fig. 8.

The basic steps involved in the Poisson surface reconstruction algorithm are:

- A 3D indicator function $\varvec{x}$ is defined in 3D space, so that its value is 1 inside the surface (the surface to be created) and 0 at points outside the surface. This indicator function needs to be approximated for reconstruction of a watertight surface.
  $$\varvec{x}(\varvec{p}) = \left\{ \begin{array}{l} 1\quad {\text{if}}\;p \subset M \hfill \\ 0 \quad{\text{if}}\;p\; \not\subset \;M \hfill \\ \end{array} \right.$$
- Gradient of the indicator function is a vector field, as $\varvec{x}$ is a piecewise continuous function. $\nabla \varvec{x}$ is non-zero only at the points near surface as those areas have changes in value of $\varvec{x}$. (Value of $\varvec{x}$ is 1 inside the surface and 0 outside. So changes in $x$ only occur near the surface).
- At the points where $\nabla \varvec{x}$ has non-zero value, the value of $\nabla \varvec{x}$ is found out to be equal to the surface normal vectors of those points. Thus the oriented normal vectors of the point samples can be taken to be the samples of $\nabla \varvec{x}$ (grad of indicator function).
- So, the problem is reduced to finding out the indicator function $\varvec{x}$, whose gradient $\nabla \varvec{x}$ is best-fit to the vector field **V** defined by the input point cloud. The equation can be expressed mathematically as:
  $$\nabla \varvec{x} = \varvec{V}$$
- To convert the above equation to a standard Poisson problem, divergence operator is applied to both sides of the equation. As divergence of gradient is Laplacian, the problem is transformed into finding out the function $\varvec{x}$, whose Laplacian equals the gradient of vector field **V**· (∇·**V**) is a function representing the surface normals at each point of the point cloud.

$$\Delta \varvec{x} \equiv \nabla \cdot \nabla \varvec{x = }\nabla \cdot {\text{V}}$$

To solve the Poisson equation, the indicator function needs to be represented in 3D space. As per the definition of the indicator function, the value of the $\varvec{x}$ needs to be accurately described near the surface, as we are interested in finding out the 3D surface near the point cloud. As can be interpreted, the value of the function away from the surface is zero, and need not be calculated far away from the point cloud. An adaptive spatial octree is used to represent the indicator function $\varvec{x}$. Each of the leaf nodes of the octree stores the values of $\varvec{x}$ at different points across the reconstructed surface.

The surface is extracted from the indicator function by using the marching cubes algorithm. An octree of predefined depth is used to store the dense point cloud. A marching cube is used to march through the 3D point cloud. The point cloud is divided into several voxel grids in the octree, as the marching cube passes through the points. The cube follows the indicator function, whose value is 1 near the surface and 0 away from the surface. After the marching cube traverse through the entire octree, triangulated 3D mesh are created by interpolating the points between the cube vertices. The 3D mesh created by the marching cubes algorithm is stored in the octree, over the point cloud.

#### Parameters affecting Poisson surface reconstruction

Poisson surface reconstruction depends upon a number of parameters. The reconstruction quality, computation time, etc. are affected by these control parameters. The influence of the parameters [as discussed in (http://www.cs.jhu.edu/misha/Code/PoissonRecon/Version7.0/ and (http://vr.tu-freiberg.de/scivi/?page_id=365)] on the reconstruction quality is discussed in the results section of this paper.

*Octree depth* Octree depth is the depth of the octree which is used during the reconstruction process. An octree of depth D produces a three dimensional mesh of resolution 2^D^ × 2^D^ × 2^D^. As the octree depth increases, mesh resolution increases. So, the memory consumption is also increased drastically. The default value of octree depth used in Poisson reconstruction is eight.

*Solver divide* Solver divide specifies the depth up to which a conjugate gradient solver is used to solve the poisson equation. Beyond this depth, Gauss–Seidel relaxation is used. If the solver divide is increased, computation time decreases as at greater value, Gauss–Seidel relaxation is used instead of gradient solver to solve the equation.

*Samples per node* Samples per node indicates the minimum number of points that is assigned at each octree leaf node by the marching cube algorithm. In case of noisy data, higher number of points are assigned in an octree, so that the surface is interpolated using all those points. This results in nullifying the effect of noise in the generated 3D surface. However, when noise free and accurate point cloud data is available, a value of 1–5 may be assigned to this variable.

*Surface offsetting* This parameter indicates a threshold correction value for the reconstructed surface. Value of 1 indicates no correction, <1 is used for internal offsetting and value of >1 is used for external offsetting.

#### Ball-pivoting algorithm for surface reconstruction

Ball pivoting algorithm (BPA) is another efficient surface reconstruction method. In BPA, a ball of predefined radius is rolled across the point cloud. As the ball traverses through the cloud, triangular interconnected 3D meshes are formed, joining the 3D points. The ball rolling is continued until all the points in the point cloud are attached to a triangle (Bernardini et al. 1999). The basic principle of BPA is discussed in two steps.

**Fig. 9:**
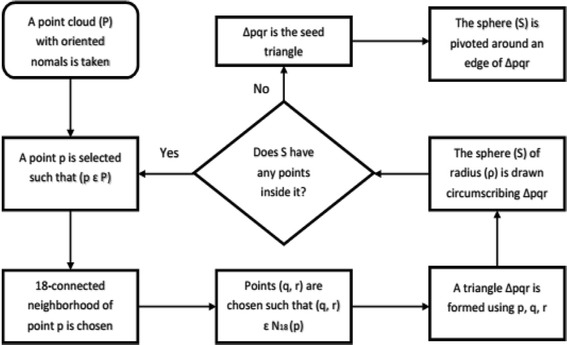
Flowchart of Ball-pivoting algorithm (Step a)
- Next, the seed triangle needs to be expanded by rolling the circumsphere, which is a ball of radius ρ. This ball is pivoted around the active edge of the seed triangle until it touches another point in the point cloud. The new-found point is joined to the endpoints of the pivoting edge of the triangle, to form another triangle. This process continues until all the points in the point cloud are traversed through and joined by triangles. This way, a 3D mesh is created over all the points in the point cloud. The procedure is described in Fig. 10.

- The first step of the algorithm, as described in Fig. 9, is to find a seed triangle inside the point cloud. This process starts with picking up a point from the point cloud. Two nearest neighbors of the point are selected and a triangle is formed using the three points. Then, the circumsphere of the triangle is checked if it has any points inside it. If the sphere does not have any point inside it, the triangle is selected as the seed triangle. Else, another point is picked from the point cloud.

**Fig. 10:**
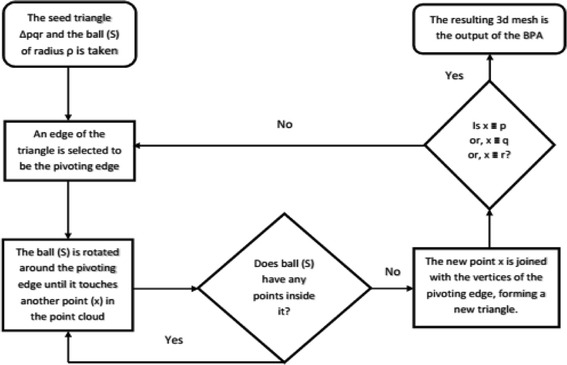
Flowchart of Ball-pivoting algorithm (Step b)

When the ball is traversed through the points, some holes are generated in the triangulated mesh, either due to the lack of points, or lack of oriented normals in the point cloud. These holes in the generated mesh are filled up by creating triangles joining the boundary edges of the holes. As BPA algorithm strongly follows the 3D points in the point cloud, presence of noise has a significant impact on the reconstructed surface. Noise handling can be done by using adaptive ball radius during the ball pivot reconstruction. BPA algorithm is data-driven, and it usually takes a longer time to generate 3D surface than Poisson reconstruction algorithm.

#### Parameters affecting Ball-pivoting algorithm

Several parameters affect the quality of the 3D surface created using BPA algorithm. These parameters are:

*Ball radius* The most important parameter of BPA is ball radius (ρ). The algorithm is very sensitive to changes in ball radius. Using a very small radius makes the model susceptible to input noise. Using large ball radius results in loss of details in the model and holes are generated in the surface. The 3D surface generated using a ball of radius ρ can’t handle a surface curvature larger than 1/ρ. This is because, the pivoting ball can’t reach the region where the curvature is higher than 1/ρ. In case of noisy data, slightly larger ball radius is encouraged, as it tends to cancel out the noise effect in the point cloud.

*Angle threshold* Angle threshold is the value of the maximum allowable angle between the active edge and the new edge created by the rolling ball. If the angle exceeds the threshold value, the ball rolling is stopped for that region. Increase in angle threshold value results in an increase in computation time.

*Clustering radius* Clustering radius is the smallest allowable distance between a newly added point to the mesh and the edge points of the active edge. If the distance between two points is smaller than clustering radius, the two points are merged together. This is done to avoid excessive memory consumption due to generation of too many small triangular meshes while processing dense point cloud.

## Results

**Fig. 11:**
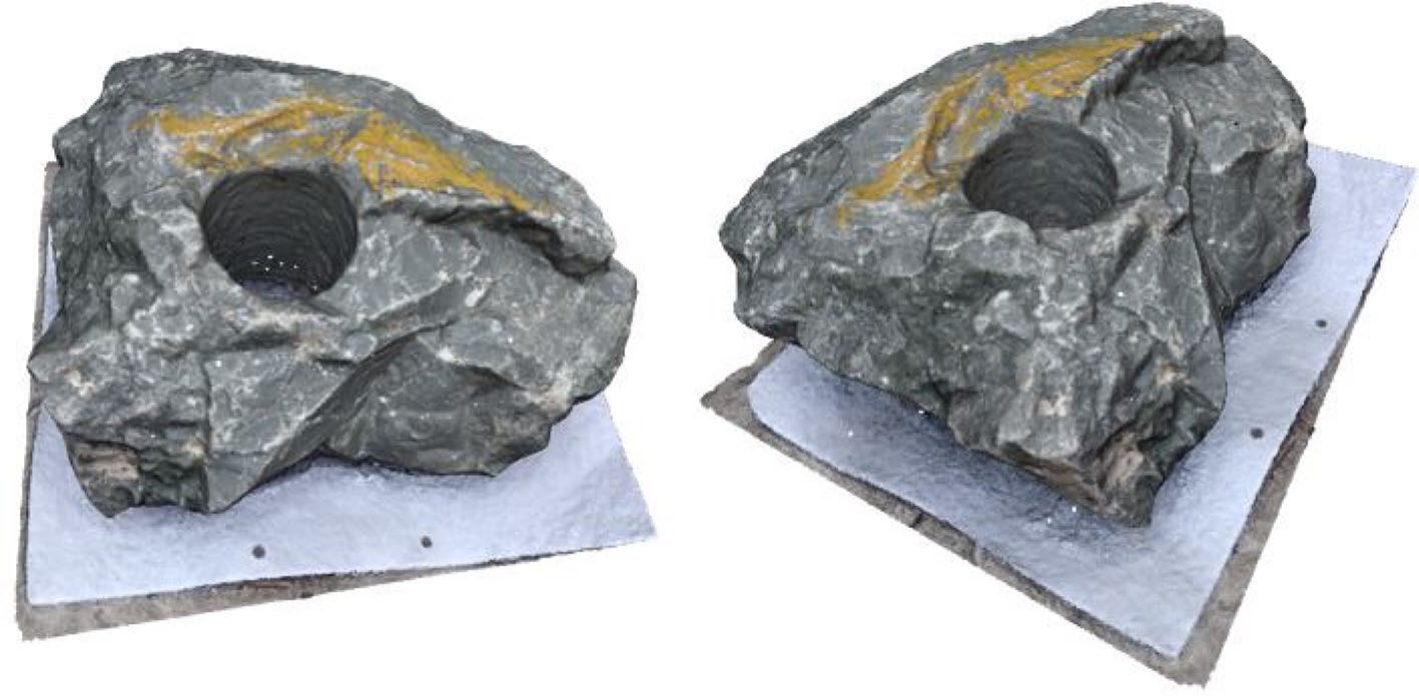
3D reconstructed textured surface of the rock-mass

**Fig. 12:**
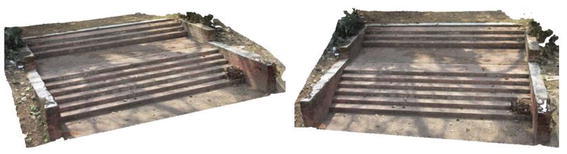
3D reconstructed textured surface of the stair case

Several point clouds were generated from digital image sequences of different geo-scenes and objects, for performance analysis of the 3D reconstruction algorithms. Poisson and Ball-pivoting surface reconstruction algorithms were implemented on the point clouds, to obtain realistic 3D surface of the captured scene or object. Different parameters affecting the surface reconstruction process during Poisson and Ball-pivoting surface reconstruction were analyzed in details, by changing the control parameters affecting the reconstruction quality. Figures 11 and 12 show the 3D reconstructed textured surfaces of a rock mass and a stair case, generated using the methodology discussed in this paper. All the results demonstrate here were obtained from a laptop having an Intel core i5-3230M dual core processor having 8 GB DDR3 RAM. Different curves in each of the graphs demonstrated in the results section, indicate changes in computation time w.r.t the control parameter being studied, while using different sets of values for the other parameters.^1^

Poisson reconstruction algorithm was used to generate 3D surfaces over a number of dense 3D point clouds. The parameters affecting the Poisson reconstruction process (as discussed in “Parameters affecting Poisson surface reconstruction” section) were changed with respect to each other and the reconstruction time, as well as the number of 3D faces created were noted down. The reconstruction quality and computation-time for the generated surfaces were used to study the effect of the said parameters, on the surface generation process.

**Fig. 13:**
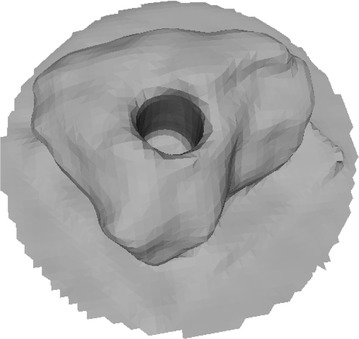
Poisson reconstruction of the rock-mass—octree 6

**Fig. 14:**
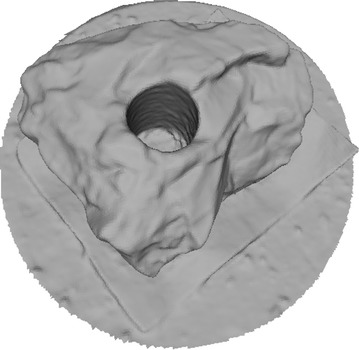
Poisson reconstruction of the rock-mass—octree 8

**Fig. 15:**
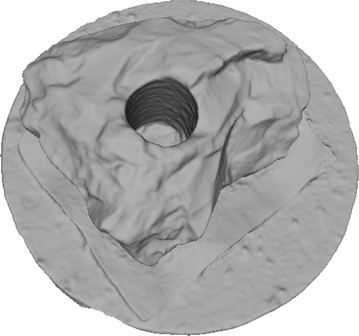
Poisson reconstruction of the rock-mass—octree 10

**Fig. 16:**
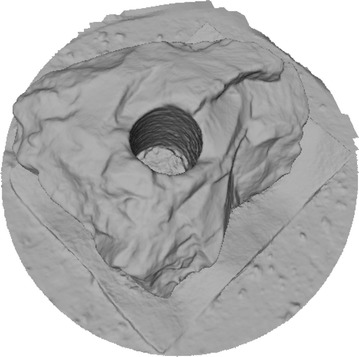
Poisson reconstruction of the rock-mass—octree 12

**Fig. 17:**
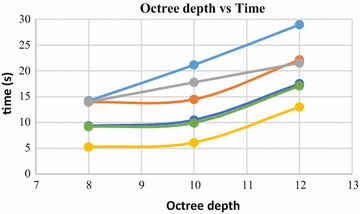
Octree depth vs computation time, 140,000 points

**Fig. 18:**
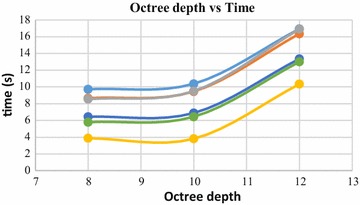
Octree depth vs computation time, 80,000 points

Octree depth was found to be one of the most important parameters in Poisson surface reconstruction. This value represents the depth of the octree being used to store the 3D surface mesh of the reconstructed object. As the octree depth increases, the resolution of the 3D mesh increases exponentially. Figures 13, 14, 15, 16 shows 3D surfaces created over a dense point cloud, using octree depths of 6, 8, 10 and 12 respectively. As can be seen from these figures, with the increase of the octree depth, quality of the reconstructed surface increases. However, as the resolution of the mesh increases, computation time also increase with the increase in octree depth value. The effect of octree depth on computation time for the surface generation process is shown in Figs. 17 and 18. The exponential increase in computation time w.r.t octree depth is expected, as octree of depth d produces a three dimensional mesh containing 2^d^ × 2^d^ × 2^d^ triangular faces. As can be seen from the equation, the mesh resolution (hence computation time) is exponentially related to octree depth d.

**Fig. 19:**
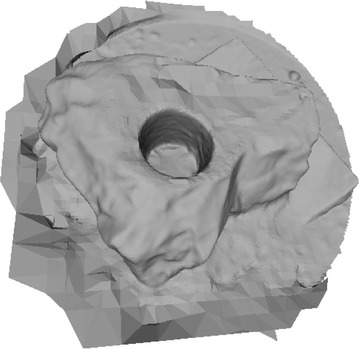
Reconstructed surface (top-sliced) of the rock-mass, SN-1

**Fig. 20:**
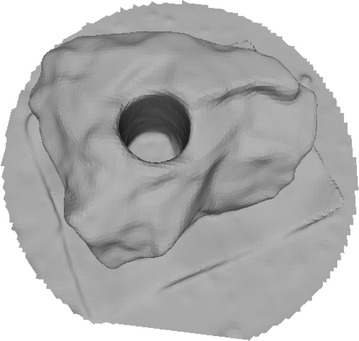
Reconstructed surface of the rock-mass, SN-6

Samples per node (SN) is the number of points assigned by the matching cubes algorithm to each octree leaf, during Poisson surface generation process. Increasing the value of SN improves the overall quality of the reconstructed surface. Figures 19 and 20 show the surface of a reconstructed rock-mass using Samples per node values of 1 and 6 respectively. As can be seen from Fig. 19, the reconstructed surface is top-spliced, when using the SN value of 1. However, using SN value of 6 solves the problem, and the regenerated surface retains the geometry of the object (Fig. 20). This can also be explained by the fact that, for higher values of SN, the marching cubes algorithm has more sample points in each octree leaf to reconstruct the surface from. However, 3D surfaces that are generated using higher values of SN, have some surface-smoothing effects (Fig. 20). Also, finer details of the surfaces are lost for very high values of SN. For point clouds having low density and noise, higher values of SN is recommended for accurate surface reconstruction.

**Fig. 21:**
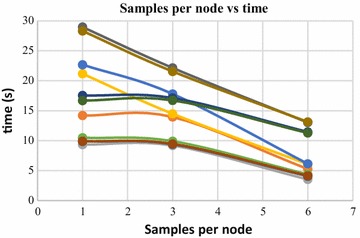
Samples per node vs time, 140,000 points

**Fig. 22:**
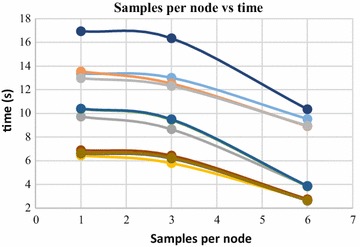
Samples per node vs time, 80,000 points

Furthermore, when the value of SN is increased, the computation time for the surface generation decreases. This is due to the fact that, with increase in value of SN, the number of 3D points assigned to each octree leaves is increased. Increased number of 3D points in an octree leaf reduces the time complexity of the operation, as there are more sample points in a leaf to interpolate the 3D surface from. Figures 21 and 22 shows the change in computation time with respect to SN values, for two dense point clouds containing 140,000 and 80,000 numbers of 3D points respectively. In these figures, the computation time is plotted against the SN values, by increasing the SN values while keeping other parameters constant. Here also, the decrease in computation time follows an exponential curve.

**Fig. 23:**
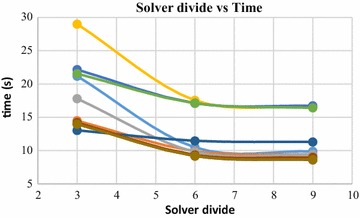
Solver divide vs computation time, 140,000 points

**Fig. 24:**
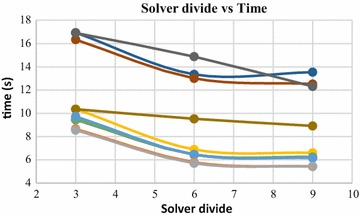
Solver divide vs computation time, 80,000 points

Solver divide is another parameter which plays an important role in Poisson surface reconstruction. This parameter specifies the depth up to which a conjugate gradient solver is used to solve the poisson equation. Beyond this depth, Gauss–Seidel relaxation is used. Increase in solver divide value leads to decrease in computation time. This trend can be observed in Figs. 23 and 24. These graphs were generated by increasing the Solver divide, while keeping the rest of the parameters constant. These figures demonstrate the variations in computation time w.r.t solver divide values, while reconstructing dense point clouds having 140,000 and 80,000 numbers of 3D points.

Ball-pivoting surface reconstruction is another successful and robust surface reconstruction algorithm from point cloud of reasonable density. In this paper, Ball-pivoting algorithm (BPA) is also used to reconstruct surface over a number of dense point clouds. Here also, the parameters affecting BPA (as discussed in “Parameters affecting Ball-pivoting algorithm” section) for surface generation is studied in details and the effects of these parameters on the reconstruction quality and computation time are discussed.

**Fig. 25:**
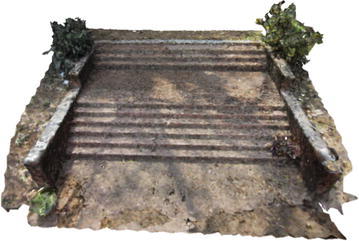
BPA obtained surface of a staircase, small ball-radius

**Fig. 26:**
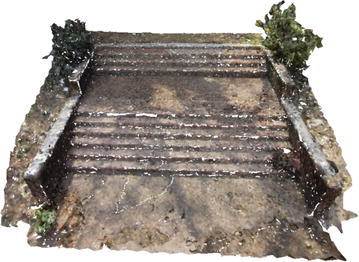
BPA obtained surface of a staircase, increased ball-radius

A staircase that has been reconstructed using BPA is shown in Fig. 25. Figure 26 is obtained by increasing the ball radius of the BPA process by 30 %. As the ball radius (BR) is gradually increased, the quality of the reconstructed surface decreases. For increased ball radius, holes begin to appear in the surface. This behavior is demonstrated in Fig. 26, where increased ball radius in BPA results in more holes in the surfaces of the stair case. This is due to the fact that, a ball of larger radius tends to ignore the 3D points nearer to its pivot triangle and jump to a point further away from its nearest neighborhood. The ignored points remain unmeshed and create holes in the model. However, for noisy point cloud, a larger ball radius is generally preferred, as the noise adjacent to the pivoting triangle gets ignored by a larger radius balls, thus a smooth noise-free surface is obtained.

**Fig. 27:**
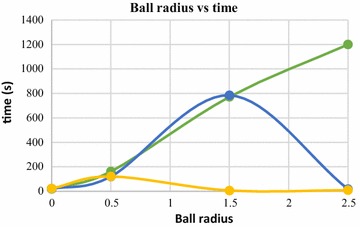
Ball radius vs computation time, 140,000 points

**Fig. 28:**
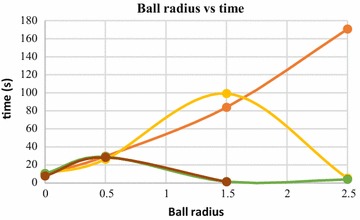
Ball radius vs computation time, 80,000 points

Computation time is significantly affected by the ball radius. Increase in ball radius up to a threshold value results in significant increase in computation time. After the threshold value of ball radius, however, the computation time decreases. This is due to the fact that, increasing the ball radius after a limiting value results in generation of holes in the 3D model, as has been shown in Fig. 26. Hole generation starts when the ball starts skipping several points in the nearest neighborhood of the pivoting edge. Due to this, the ball traverses less number of points and computation time decrease. However, choosing the ball radius higher than the threshold value is not recommended. When the ball radius value exceeds the threshold value, degradation of the reconstruction quality takes place, due to presence of holes in the surface. The relation of computation time with ball radius is graphically explained in Figs. 27 and 28. These graphs were obtained by increasing the ball radius gradually, while BPA operation was carried out on two dense point clouds having 140,000 and 80,000 3D points.

Clustering radius (CR) is another important parameter, which affects the quality of the surface reconstruction in BPA. CR is usually expressed in terms of percent of ball radius (BR). This distance denotes the minimum allowable distance between two points in the 3D mesh. If two points in the meshed point cloud are closer than CR, they are merged together to form a common vertex. The merging is done to avoid excessive memory consumption in generating and storing the 3D mesh from a dense point cloud. Increase in CR results in decrease of computation time. This is because, larger CR value results in merging of more points in the point cloud, thus decreasing the number of available points for the ball to traverse through. This trend can be visualized in Fig. 34, where computation time is plotted against increasing CR values.

**Fig. 29:**
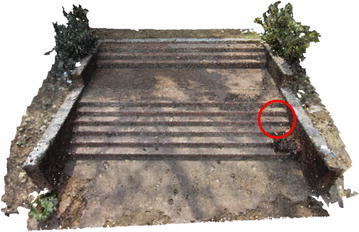
BPA generated surface using CR value = 5 % of BR

**Fig. 30:**
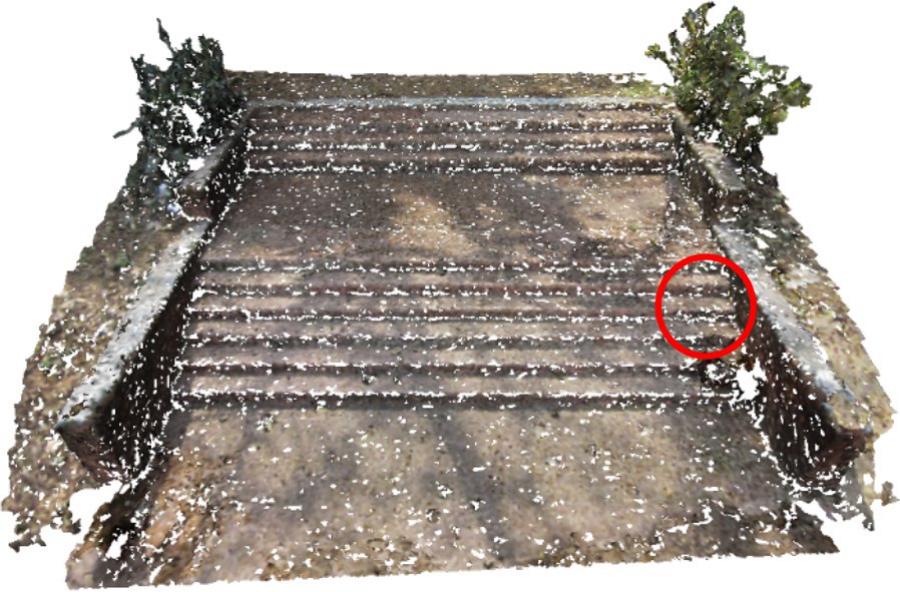
BPA generated surface using CR value = 30 % of BR

However, due to the excessive merging of adjacent 3D points in the point clouds, larger CR results may result in incomplete surface generation with holes. Also, sometimes, use of higher CR value results in 3D surfaces where important features of the object is lost. This behavior can be seen from Figs. 29 and 30. Use of larger CR during BPA process results in loss of the flower tub from Fig. 30, whereas the tub is present in Fig. 29, when a smaller CR value is used. The flower tub being discussed, is highlighted in Figs. 29 and 30. Also, the edges of the staircase in Fig. 30 is broken and the overall quality of the reconstruction is bad as large CR value was used during the reconstruction.

**Fig. 31:**
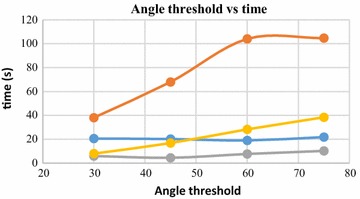
Angle threshold vs computation time

**Fig. 32:**
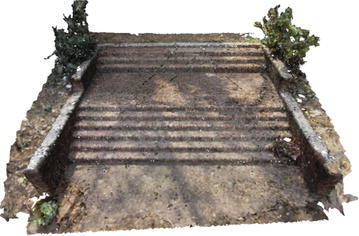
BPA using angle threshold of 90°

**Fig. 33:**
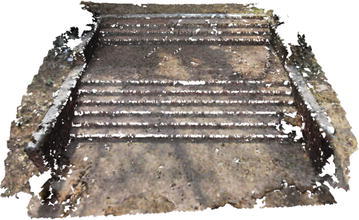
BPA using angle threshold of 30°

Angle threshold is the value of the maximum allowable angle between the active edge and the new edge created by the rolling ball. If the angle exceeds the user defined threshold value, new edge creation is stopped and the corresponding vertex is omitted by the ball. This is done to stop creation of triangular mesh with high obtuse angles. Increase in angle threshold value results in increase of computation time, as more edges are added to the 3D mesh by the rolling ball. This trend is visible from Fig. 31. However, use of smaller values of the angle threshold leads to omission of mesh creation in a lot of points. This results in loss of details in the reconstructed surface. Figures 32 and 33 represent two reconstructed surfaces, having angle threshold values of 90° and 30° respectively. In Fig. 33, a lot of edges were stopped from being created by BPA, as the angle made by the new edge with the pivoting edge exceeded 30°. Predictably, the surface in Fig. 33 has suffered from loss of details.

## Conclusion

This paper presents a 3D reconstruction framework, which is used to reconstruct photo-realistic 3D watertight surfaces of regular and irregular shaped objects, using digital images of the objects. Here, digital images of geo-objects were used to obtain sparse, followed by dense 3D point clouds of the objects. These 3D point clouds (having oriented normal, and RGB color information for each points) were used for generation of 3D watertight surface of the objects. Poisson surface reconstruction and Ball-pivoting algorithm for surface reconstruction were used to generate accurate and photo-realistic 3D surface over the dense point clouds. Performance analysis of these two reconstruction algorithms were done by studying the effects of changing the control parameters, on the quality of reconstructed 3D surface. Some of the key aspects of this study are discussed below.

### A simplistic 3D reconstruction approach

A methodology has been discussed, using which 3D reconstruction of a geo-object or scene can be done using digital image sequence of the object. Dense point clouds, obtained from correspondence point matching across the digital image sequence of an object, is used to generate photo-realistic 3D surface of the object. The dense 3D point clouds are processed using two existing surface reconstruction algorithms: Poisson Surface reconstruction algorithm and Ball-pivoting algorithm for surface reconstruction. Later, photo-realistic texture integration was done over the generated 3D surfaces to generate complete 3D model of the objects being studied.

The 3D reconstruction methodology described and used here is based on several previously established algorithms in fields of correspondence matching, structure-from-motion, surface reconstruction etc. Rather than going into detailed mathematics involved in development of these algorithms, the authors concentrated on applying these existing algorithms to develop a cost-effective, robust and simplistic solution to the 3D reconstruction problem. The approach mentioned in this paper can be readily used in industries due to its robustness, speed and ability to reconstruct complex geo-objects with highly irregular geometry. With its low manpower requirement and almost zero operating cost, the photogrammetry based 3D reconstruction approach discussed here can effectively replace the expensive laser-scanners as primary 3D mapping methods in industries.

### Poisson surface reconstruction and its control parameters

Poisson surface reconstruction is one of the algorithms which was used to generate 3D surface from dense point clouds obtained using digital images of the objects. The effect of the control parameters of Poisson surface reconstruction on quality of 3D surface is summarized below.

- *Octree depth* Octree depth used during the Poisson surface reconstruction plays a key role in surface generation from dense point cloud. Increase in octree depth results in high resolution 3D mesh, thus improving the overall quality of the surface (Figs. 13, 14, 15, 16). This fact can be directly inferred from studying the Poisson reconstruction algorithm, as the number of faces in a surface of octree depth d is 2^d^ × 2^d^ × 2^d^. On the downside, surface generation time and CPU resource usage drastically increase with the increase in Poisson reconstruction octree depth.
- *Samples per node* Samples per node is another important parameter affecting the quality of the 3D surface generated using Poisson reconstruction algorithm. Increasing the samples per node value adds more 3D points in each octree leaf. As a result, the computation time decreases with increase in this parameter (Figs. 21, 22). With the increase in the samples per node value, marching cubes algorithm can use more points in each octree grid point while reconstructing the surface (Figs. 19, 20). So, noisy and low density point cloud can be reconstructed accurately using larger values of samples per node.

### Ball-pivoting algorithm for surface reconstruction and its control parameters

Ball-pivoting algorithm (BPA) for surface reconstruction was also used to generate 3D surface of the objects being reconstructed, in this article. The effects of the control parameters of BPA on quality of generated 3D surface is summarized below.

**Fig. 34:**
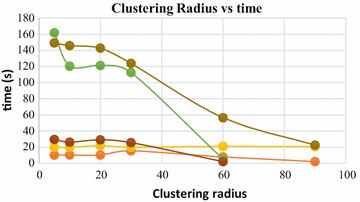
Clustering radius vs computation time
- *Angle threshold* Angle threshold value used in BPA for surface generation is also important. The quality of the reconstructed surface improves with increase in angle threshold value. But, increase in angle threshold value leads to increase in computation time (Fig. 31). An angle threshold value of 90° is generally preferred in a dense and noise free point cloud. Using smaller values of angle threshold leads to creation of holes in the 3D surface (Fig. 33).

- *Ball radius* The quality of the reconstructed surface depends heavily on the ball radius. Increasing the ball radius causes loss of details and creation of holes in the reconstructed surface (Figs. 25, 26). Computation time also increase with increase in ball radius value, up to a certain threshold radius. After the thresholding ball radius, increase in the radius causes the computation time to decrease exponentially, although the reconstruction quality goes down drastically as we go beyond the thresholding ball radius (Figs. 27, 28). A slightly larger ball radius is generally preferred in case of noisy and low density point clouds.
- *Clustering radius* Clustering radius is another parameter, which plays a key role in determining the quality of surface generation in BPA. When the value of this parameter is increased, the computation time for surface generation decreases (Fig. 34). However, increase in clustering radius causes loss of important details in the 3D model being reconstructed, as merging of adjacent 3D point of the point cloud take place when larger values of clustering radius are used (Fig. 30).

## Additional file

10.1186/s40064-016-2425-9 Datasheet.

## Footnotes

1 The datasheet of the generated graphs is submitted as a supplementary excel sheet (Additional file 1).
